# White Blood Cell Classification Using Texture and RGB Features of Oversampled Microscopic Images

**DOI:** 10.3390/healthcare10112230

**Published:** 2022-11-08

**Authors:** Furqan Rustam, Naila Aslam, Isabel De La Torre Díez, Yaser Daanial Khan, Juan Luis Vidal Mazón, Carmen Lili Rodríguez, Imran Ashraf

**Affiliations:** 1 School of Computer Science, University College Dublin, D04 V1W8 Dublin, Ireland; 2 Department of Software Engineering, University of Management and Technology, Lahore 544700, Pakistan; 3 Department of Computer Science, School of Systems and Technology, University of Management and Technology, Lahore 54770, Pakistan; 4 Department of Signal Theory and Communications and Telematic Engineering, University of Valladolid, Paseo de Belén 15, 47011 Valladolid, Spain; 5 Higher Polytechnic School, Universidad Europea del Atlántico, Isabel Torres 21, 39011 Santander, Spain; 6 Department of Projects, Universidad Internacional Iberoamericana (UNIB), Arecibo, PR 00613, USA; 7 Department of Project, Universidade Internacional do Cuanza (UNIC), Barrio Kaluanda, Cuito EN 250, Angola; 8 Department of Project Management, Universidad Internacional Iberoamericana, Campeche 24560, Mexico; 9 Department of Information and Communication Engineering, Yeungnam University, Gyeongsan 38541, Korea

**Keywords:** white blood cells classification, leukemia, texture features, Chi-squared, SMOTE

## Abstract

White blood cell (WBC) type classification is a task of significant importance for diagnosis using microscopic images of WBC, which develop immunity to fight against infections and foreign substances. WBCs consist of different types, and abnormalities in a type of WBC may potentially represent a disease such as leukemia. Existing studies are limited by low accuracy and overrated performance, often caused by model overfit due to an imbalanced dataset. Additionally, many studies consider a lower number of WBC types, and the accuracy is exaggerated. This study presents a hybrid feature set of selective features and synthetic minority oversampling technique-based resampling to mitigate the influence of the above-mentioned problems. Furthermore, machine learning models are adopted for being less computationally complex, requiring less data for training, and providing robust results. Experiments are performed using both machine- and deep learning models for performance comparison using the original dataset, augmented dataset, and oversampled dataset to analyze the performances of the models. The results suggest that a hybrid feature set of both texture and RGB features from microscopic images, selected using Chi2, produces a high accuracy of 0.97 with random forest. Performance appraisal using k-fold cross-validation and comparison with existing state-of-the-art studies shows that the proposed approach outperforms existing studies regarding the obtained accuracy and computational complexity.

## 1. Introduction

White blood cells (WBC) are potent infection fighters; they normally grow and divide in an orderly way regarding the needs of the human body to fight infections and other diseases [1]. However, in people with leukemia, the bone marrow produces an excessive amount of abnormal WBCs that do not function properly, and this leads to several abnormalities [2,3]. The counts of WBCs in blood cells can provide early clues of different probable abnormalities concerning the number of cells of different types of WBC. The WBCs can be categorized into five major classes: lymphocytes, eosinophils, neutrophils, basophils, and monocytes. The percentage of each type of WBC in a healthy person varies within a range. For example, lymphocytes are 20% to 40%, eosinophils account for 1% to 6%, and monocytes make 2% to 10%, while neutrophils are 40% to 80% [4]. A lower count of WBC can cause blood cancer and many other diseases, as WBC contributes as an important part of the body’s immune system. The number of WBCs varies when the bone marrow stops making them, or when WBC are destroyed by another entity [4]. Healthy WBCs play an important role in preventing different infections and helping to fight other deadly diseases such as COVID-19 [5].

Estimating the WBC can help with making an early prediction of probable diseases. Blood microscopic images can be used to detect the WBC type and the timely diagnosis of the disease. Traditional approaches for WBC-type detection are time-consuming and low in accuracy, which increases the importance of accurate systems for the fast and accurate analysis of WBC. In this regard, the machine learning approach plays an important role. Several machine learning approaches are introduced recently, which can predict the type of WBC using microscopic images. These include the study [6] using convolutional neural networks (CNN) and an extreme learning machine (ELM) for WBC type detection using microscopic images. CNN with traditional deep learning approaches and handcrafted features for WBC is deployed by the study [7].

This study also follows a machine learning-based approach for WBC type detection using blood microscopic images. We used a WBC image dataset acquired from the IEEE data port, which has imbalanced class distribution and a poor feature set. We proposed a novel approach by combining a data re-sampling technique and a hybrid feature engineering technique to alleviate the influence of such shortcomings and to make the following contributions.

- An improved feature set is obtained by combining the texture features and RGB features to make a more correlated feature set with target classes to obtain a high accuracy. Later, Chi-squared (Chi2) is used to select an important and equal number of features for the models’ training. The models are evaluated using texture features and RGB features, in comparison to the proposed hybrid features.
- The imbalanced distribution of different classes of WBC is tackled using data resampling, which helps to reduce the model over-fitting. The synthetic minority oversampling technique (SMOTE) is applied for data resampling in this study for evaluating the influence of data resampling on the performance of machine learning models.
- Besides using various machine learning models such as decision tree (DT), random forest (RF), k-nearest neighbor (KNN), and support vector machine (SVM), state-of-the-art pre-trained deep learning models are also employed, including ResNet50 and VVG16. In addition, a custom-designed CNN is also used. Experiments are performed using the original dataset, augmented dataset, and oversampled dataset.
- The performances of all models is analyzed regarding different performance evaluation metrics. Furthermore, k-fold cross-validation and statistical *t*-tests are carried out. Additionally, the performance comparison with recent state-of-the-art approaches is made to analyze the performance of the proposed approach regarding accuracy and response time.

This paper is further divided into three sections. Section 2 discusses several important studies in the context of WBC-type detection. The proposed feature engineering approach and the processes of WBC-type classification are explained in Section 3. Section 4 presents the results and discussion, while the conclusions are provided in Section 5.

## 2. Related Work

Research on WBC is one of the most important domains in bioinformatics, while the use of machine learning models for WBC has also been regarded as potentially significant [8,9,10]. The classification of WBC using microscopic images has been investigated by several researchers [6,7,11]. Despite that, several challenges remain unresolved, such as the pure WBC image dataset, the high accuracy machine learning approach, the efficiency and reduction in computational time, etc. In the following, several prominent research works are discussed.

The authors propose a deep learning-based automatic approach for WBC classification in [9]. The study utilizes pre- and post-preprocessing to improve the performance of the CNN model. In preprocessing, data normalization, filled holes, and data augmentation are applied, while in post-preprocessing, the study deployed localization and data segmentation techniques. The study achieved a 95.73% accuracy score using the CNN model on the cytological images dataset. The study [12] used the CNN model and recurrent neural network (RNN), as well as, a combination of both CNN and RNN for WBC classification to resolve the multiple cells overlap problem. The Canonical Correlation Analysis method is used in the study with the BCCD dataset. The achieved accuracy score is 95%. The study [13] proposed a 3D convolutional network, called deep hyper, for WBC microscopic image classification. Spectral and spatial features are used with a deep hyper model to obtain 96% accuracy. Using the deep hyper model with an attention module resulted in a 97% accuracy score.

The study [14] used a hybrid approach for WBC detection, in which the scale-invariant feature transform (SIFT) and CNN model are combined. SIFT is used for feature detection, which is used for CNN training using the LISC and WBCis datasets. The proposed model achieved 95.84% and 97.33% accuracy scores, respectively, for both datasets. Similarly, the study [15] proposed an approach for the classification of WBC using the CNN model. They deployed the proposed approach on the Kaggle WBC images dataset and achieved significant accuracy. The study [16] proposed a multi-level CNN model for the WBC classification for four types of cell classification. At the first level, Faster R-CNN is applied for the detection of the region of interest while at the second level, CNN-based architecture MobileNet is used for cell-type classification.

Besides proposing novel architectures, several studies adopt pre-trained models for WBC classification. The study [6] used a supervised machine learning approach for WBC detection which follows a CNN architecture and ELM model on a microscopic image dataset. Several CNN-based pre-trained models are used, such as AlexNet, GoogleNet, VGG-16, and ResNet for feature extraction while ELM is trained on those features to obtain a 96.03% accuracy. Similarly, [7] used deep learning CNN with handcrafted features to achieve a higher accuracy. The study worked on six types of WBC, including lymphocytes, monocytes, basophils, neutrophils, eosinophils, and abnormal cells. The experimental results are promising. Along the same directions, [17] used a pre-trained CNN architecture for WBC classification. The authors deployed ResNet and Inception variants with fine-tuned parameters to obtain 100% training accuracy with the ResNet50 model for four classes of WBC; however, the computational complexity of this approach is very high, as it uses 3000 epochs for a very deep ResNet50 model. The study achieved an accuracy score of 98.4%. The study [18] used the DenseNet121 model for the classification of the WBC. Data normalization and data augmentation are used with the optimized DenseNet121 model. The proposed model achieved 98.84% accuracy on the KBC dataset.

Table 1 presents a comparative analysis of the cited research works. Predominantly, the above-discussed studies on WBC classification adopt deep learning approaches where the computational costs are higher than for machine learning models. Additionally, several studies use a lower number of WBC classes, and the reported accuracy is high. In addition, a few studies experimented with imbalanced datasets where the models’ over-fitting probability is high. This study resolves the problem of high computational cost by deploying machine learning models, and aims at achieving high accuracy using feature engineering. Using a balanced dataset, the probability of model over-fitting is also reduced.

## 3. Materials and Methods

This study works on WBC type detection using the image dataset and machine learning approach. In the following sections, the background on WBC, details of the dataset, and a description of feature engineering and models are provided.

### 3.1. White Blood Cells

WBC are colorless cells of the human blood because they do not have any pigment. They comprise 7000 to 8000 cells in one cubic mm of blood [4]. Their size is much larger than the red blood cells. They can be classified into five different types, based on the shape of the nucleus and the density of the granules in the cytoplasm. WBCs have a single large bilobed nucleus, which distinguishes them from the other blood cells. They are formed in the bone marrow cell and then move to the blood and lymph [4]. Types of WBC and their brief description are provided here.

**Figure 1:**
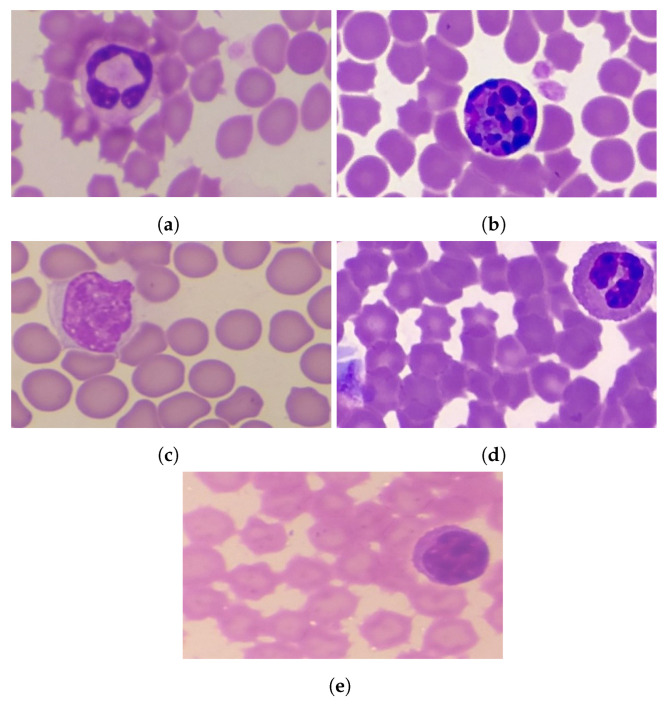
Types of WBC. (**a**) Neutrophils, (**b**) Basophils, (**c**) Eosinophils, (**d**) Monocytes, and (**e**) Lymphocytes.

- Neutrophils make up about 62% of the total WBC. They can engulf any foreign particles such as viruses and bacteria, and destroy them to neutralize their effects. They are about twice the size of a red blood cell. Their nucleus contains about 2 to 5 lobes [21]. Figure 1 presents the types of WBC.
- Basophils comprise approximately less than 1% of WBCs. They are about twice the size of a red blood cell, with a bilobed nucleus. These cells release the protein called heparin, which prevents blood clotting. Basophils also release another protein called histamine, which causes inflammation. They release antibodies and antibiotics, which protect the body from the effects of any foreign objects. Figure 1b shows the types of basophils of WBCs Figure 1b.
- Monocytes forms approximately 3% of the total WBC. They are about two to three times larger than red blood cells. The nucleus is an almost round to lobed shape. It produces macrophages, which can destroy the larger particles via phagocytosis. The life span of macrophages in the blood is about 8 to 10 h, after which they move toward the lymphoid tissue and become a macrophage. The monocyte type of WBC is shown in Figure 1c.
- Eosinophils comprise about 2% of the WBC. They are about twice the size of a red blood cell, while their nucleus is bilobed. They inactivate the inflammation-producing substance, and attack parasites and worms. Figure 1d shows the sample image of the basophil types of WBC.
- Lymphocytes make up approximately 32% of the total WBCs (Figure 1b). Their size is nearly equal to the red blood cell. They produce antibodies. The life span of lymphocytes in the blood may be months and even years, depending upon the activity of the cell. Lymphocyte samples are shown in Figure 1e.

**Figure 2:**
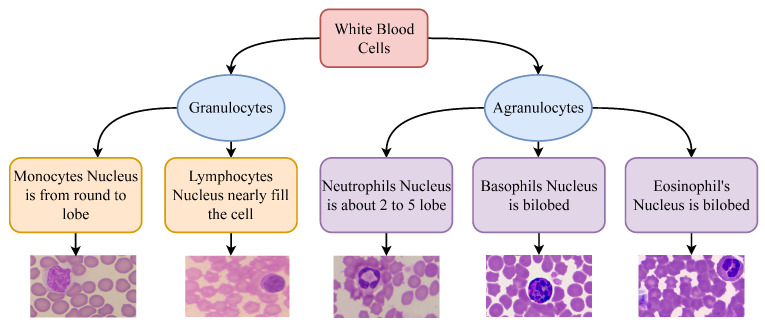
Hierarchy and classification of WBCs.

Figure 2 shows the hierarchy of the WBC type according to the root types, and also illustrates the differences in various types of WBC.

### 3.2. Flow of Implemented Methodology

In this study, first, a dataset containing the WBC images is acquired from the IEEE data port, which is later used to extract the features from the WBC images. We extract two types of features, including texture features and RGB features. Texture features are extracted using the skimage library [22]. After extracting the features, we deploy the feature selection technique to extract the prominent and most important features using Chi2.

**Figure 3:**
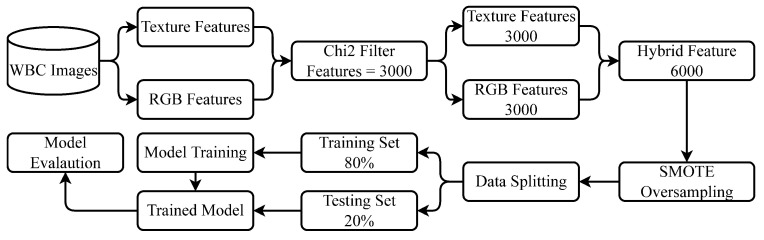
Flow of the proposed methodology.

We select 3000 features each from the RGB and texture features. Both types of important features (RGB and texture) are combined to form a hybrid feature set. For resolving the problems related to the imbalanced dataset, we performed data oversampling using SMOTE, which helps to reduce the model over-fitting towards the majority class. Several machine learning and deep learning models are deployed to perform WBC-type classification. To train and test the learning models, we split the dataset into training and testing sets with 0.8 to 0.2 ratios for training and testing, respectively. In the end, we evaluate all models in terms of accuracy, precision, recall, and F1 score. Figure 3 shows the proposed methodology of this study.

### 3.3. WBC Dataset

The dataset that is used in this study comprises five categories of WBCs, including neutrophil lymphocyte, monocyte, eosinophil, and basophil. The dataset is acquired from the IEEE Dataport [23]. Each type of WBC consists of a different number of images, as shown in Table 2. The total number of images in the dataset is 3539, with 667 raw images, 1464 augmented images, and 1408 cropped and classified images.

### 3.4. Feature Engineering

For feature engineering, we deployed feature extraction and feature selection techniques to generate an important feature set that can enhance the performance of learning models. We extracted two types of features from the dataset, which are RGB features and texture features, because color and shape are the best parameters for classifying the WBC images.

#### 3.4.1. RGB Features

Color is an important part of the image, and is widely used in image processing. The color information is conveyed through three channels, and these color features are invariant to the rotation of the pixels in an image. The red, blue, and green pixel values represent the content in the image, and can be used for the training of machine learning models [24]. These RGB features are very feasible, as color can potentially be used to discriminate between images of the different target classes.

#### 3.4.2. Texture Features

Texture features can be extracted from grayscaled images, disregarding the color information [25]. Texture features provide information about the intensities of the pixels in an image. They are characterized by the distribution of intensity levels in the neighborhood.

Both RGB and texture features have their own advantages and disadvantages, similar to their significance toward improving the performances of machine learning models. For RGB features, we used the CV2 library, while the texture features are obtained using the skimage library [22]. Table 3 shows the number of features for RGB and texture features.

We combined both features vertically and generated a hybrid feature set. Before combining the features, we performed feature selection and extracted the most important features, which reduced the size of the hybrid feature set to improve the accuracy and efficiency of the models. For feature selection, we used the Chi-squared (Chi2) technique.

#### 3.4.3. Chi2

Chi2 is a feature selection technique that finds the relationship between the feature set and the target classes [26]. It finds the dependence of the target class on each of the data features. It assumes the null hypothesis, indicating that the feature distribution is independent. Chi2 calculates the $x^{2}$ scores and then orders them in descending order. The higher the value of $x^{2}$, the more important the feature is. The formula to calculate the $x^{2}$ is given as (1)

(1) $$x^{2}=\sum_{i=1}^{k}\sum_{j=1}^{m}\frac{{\left(O_{ij}-E_{ij}\right)}^{2}}{E_{ij}}$$

where $O_{ij}$ is the observed frequency, $E_{ij}$ is the expected frequency, *k* is the number of rows and *m* is the number of columns, while (i,j) denotes the cell.

**Figure 4:**
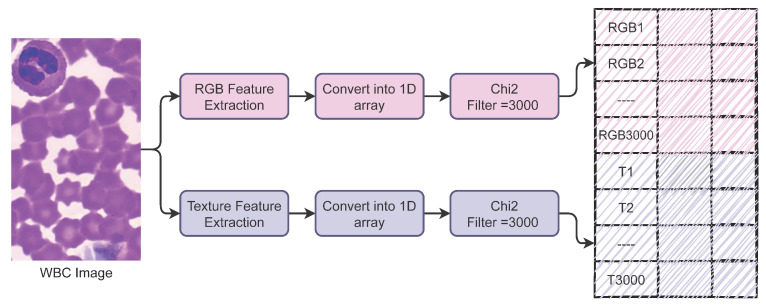
Process followed to make the hybrid feature set.

We extract 3000 features each from RGB and texture features and make a hybrid feature set. Table 4 shows the number of features with each approach, and Figure 4 shows the process of forming the hybrid feature set.

### 3.5. Synthetic Minority Oversampling Technique

**Figure 5:**
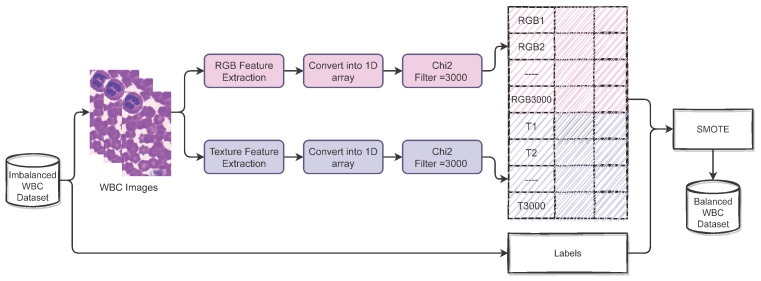
Approach followed for data balance using SMOTE.

SMOTE is an oversampling technique that generates artificial samples to make a balanced dataset that can help to reduce the probability of models overfitting [27]. SMOTE generates samples for the minority class to equal the number of majority class samples. It is mostly used for the numeric data features, while this study uses it to increase the number samples for the image data. Table 5 shows the ratio of samples after the oversampling of data. Figure 5 shows the approach for data normalizing and balancing using the SMOTE approach.

### 3.6. Data Splitting

Data splitting is performed to train and test the learning models. We split the dataset with 0.8 to 0.2 ratios into cases of both oversampling and the original dataset. This ratio is more optimal as the dataset size is not too large, so we obtain the best results with this ratio. Table 6 shows the number of samples for training and testing with the original dataset and the over-sampled dataset.

### 3.7. Machine Learning Models

Machine learning models can be used to solve different domain problems such as text analysis [28], computer vision application [29,30], IoT [31,32], and image processing [33,34], etc. In this study, we used machine learning models for WBC image classification. We deployed several machine learning models on hybrid features set to show the significance of our feature engineering approach. Four state-of-the-art machine learning models such as DT, RF, KNN, and SVM have been employed with their best hyper-parameters settings according to the dataset used, as shown in Table 7.

DT is a tree-based machine learning model used for classification and regression tasks [35,36]. DT constructs a tree using the features set, and put important features on the root nodes, while leaf nodes function as decision nodes for the DT. The features’ importance in DT can be calculated using the Gini Index or Information Gain algorithms. These algorithms provide purity and impurity in the feature set. For tree split, we used the Gini criterion [37], which can be calculated as

(2) $$G=\sum_{i=1}^{T}p\left(i\right)*\left(1-p\left(i\right)\right)$$

where *T* is the total number of target classes and p(i) is the probability of picking the data point with class *i*.

The DT is used with max_depth parameter with value 200, which is the most optimal value for it, because after a 200 value of max_depth, there is no change in model accuracy. The increase in tree depth increases the complexity of the model, so we restrict it to 200-level depth.

RF is also a tree-based model with an ensemble of several DTs. RF can be used for classification and regression tasks. It can perform well on nonlinear and imbalanced datasets [38]. RF combines a number of DTs under majority voting. All DTs in RF make their prediction on the test data, and then the most predicted class by the DTs is regarded as the final prediction by the RF. RF can be defined mathematically as

(3) $$rf=mode\{DT_{1},DT_{2},DT_{3},...,DT_{n}\}$$

or,

(4) $$rf=mode\left\{\sum_{i=1}^{n}DT_{n}\right\}$$

where $DT_{1},DT_{2},DT_{3}$ are the decision trees and *n* is the number of decision trees.

We used RF with n_estimator parameters with 200 and max_depth parameters with 200 as we used in DT. The n_estimators indicate the number of DTs participating in the prediction procedure of RF.

KNN is a simple machine learning algorithm for classification and regression. KNN, also known as lazy leaner, uses the whole training data. It performs matching on test data with training data, and finds the distance with the nearest target in the training data [39]. Several distance metrics can be used with KNN, while the Euclidean distance is most commonly used:

(5) $$d\left(i,j\right)=\sqrt{\sum_{p=1}^{n}{\left(i_{p}-j_{p}\right)}^{2}}$$

where *i* and *j* are two points in Euclidean *n*-space, and $i_{p}-j_{p}$ shows the Euclidean vectors, starting from the origin of the space.

KNN is used with only one parameter, which is n_neighbors with a value of 5, indicating that the model uses five neighbors to predict the class of a given sample.

SVM is a linear model used for classification and regression tasks. SVM draws multiple hyper-planes to classify the data into several classes [40]. Hyper-plane with the best margin from data points will be the selected hyper-plane for the SVM. Hyper-plane can be defined mathematically as

(6) Z.x+b=0

where *Z* is a vector normal to hyper-plane and *b* is an offset. We used SVM with poly kernel and C = 4.0.

### 3.8. WBC Classification

Algorithm 1 illustrates the WBC classification approach used in this study. The given input image is classified into one of the five classes. First, RGB and texture features are extracted from all images one by one, and then concatenated vertically (axis = 1) to make a hybrid feature set. The feature set goes through the SMOTE technique to make the dataset balanced, which is later used to train machine learning models.
**Algorithm 1:** WBC classification algorithm**Input:** WBC Images**Result:** WBC type detection ⟶ (Neutrophil Lymphocyte, Monocyte, Eosinophil, and Basophil)initialization;**loop** (Img in Images)               RGB $⟵R_{f}$(Img)               Txt $⟵T_{f}$(Img)               $R_{f}.append\left(RGB\right)$               $T_{f}.append\left(Txt\right)$**loop end**HF $⟵concatenate(R_{f},T_{f}axis=1)$PFS ⟵SMOTE(HF)Prediction $⟵ML_{model}\left(PSF\right)$Scores ⟵Evaluation(Prediction)

## 4. Results

This section contains the results of machine learning and deep learning models for WBC-type detection. We deployed DT, RF, KNN, and SVM from machine learning models with both texture and RGB features. We used a Core i7 11th generation machine with NVIDIA GPU to perform the experiments. The system consists of 16 RAM and 1TB SSD. We used a Jupyter notebook and Python language to implement the approach.

### 4.1. Results Using the Original Dataset

Table 8 contains the results of machine learning models on the original dataset with texture and RGB features. The results of all models are very poor on the original dataset because the target ratio in the original dataset is highly imbalanced. All models show the over-fitting for the majority class data. With both the RGB and texture features, the performance of RF is better as compared to other models in terms of accuracy on the original dataset, as it achieves 0.66 and 0.62 accuracy scores, respectively. The F1 scores of all models are poor because of the highly imbalanced dataset. To resolve this imbalanced dataset problem, we used data augmentation.

### 4.2. Results Using the Augmented Dataset

For data augmentation, ref. [23] used Keras preprocessing layers, where the images are augmented to generate a larger dataset. The data augmentation influences the learning models positively and marginally improved the accuracy. The marginal improvement is due to the uncorrelated distribution of features during augmentation. Table 9 contains the results of machine learning models on the augmented dataset with both the texture and RGB features. For the augmented dataset, machine learning models with RGB features are somehow better as compared to the texture features, as RF achieves a 0.64 accuracy score with a 0.54 F1 score. With texture features, RF achieves a 0.35 F1 score. The reason behind the accuracy improvement of the learning models with RGB features after augmentation is that the WBC color is not changed after augmentation, while the texture features are changed after flipping or rotating the image during augmentation.

### 4.3. Performance of Models on Hybrid Features

We try to improve the performance of learning models by making a balanced dataset using the SMOTE technique. The SMOTE technique yields a highly balanced dataset, which increases the number of samples for training and testing for machine learning models and results in significant improvements. Table 10 shows the results of machine learning models using the SMOTE balanced dataset. Using the balanced dataset with SMOTE, the performances of models has been elevated, as the RF achieves the highest accuracy score of 0.92 and the same F1 score using the texture features. SMOTE does not impact on the texture features and the RGB features, as it generates new samples artificially to reduce the models’ over-fitting. RF is also good with RGB features after oversampling with SMOTE, as it achieves a 0.87 accuracy score. DT and KNN do not perform well because KNN shows a poor performance when used with a large feature set.

A novel hybrid feature engineering approach is deployed with the machine learning models to analyze their performances against the individual texture and RGB features. The hybrid features set combines both the texture features and RGB features selected using the Chi2 technique. In addition, SMOTE is applied to perform the oversample for data balancing.

Table 11 shows the results of machine learning models using the hybrid feature engineering technique. The results suggest that this approach significantly improves the performance of all learning models, with RF being the highest accuracy-preserving model, as it obtains an accuracy score of 0.97. The performance of RF is better, owing to its ensemble architecture which combines 300 decision trees on the hybrid feature set to make the final prediction. SVM also shows significant performance with the hybrid feature, as it achieves a 0.89 accuracy score and a 0.89 F1 score. The impact of the hybrid feature set is two-fold, where it provides a highly correlated feature set in the first place and a balanced feature set in the second place, which improves the training process of the machine learning models and enhances their performances. On average, the performances of all the models have been elevated when used with the hybrid feature set.

**Figure 6:**
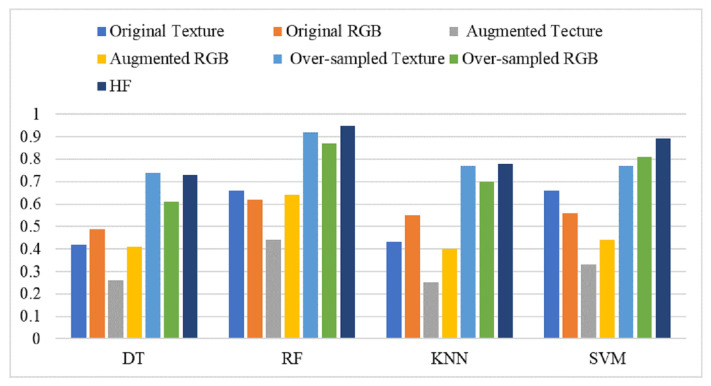
Comparison between all machine learning approaches used for classification of WBC types.

**Figure 7:**
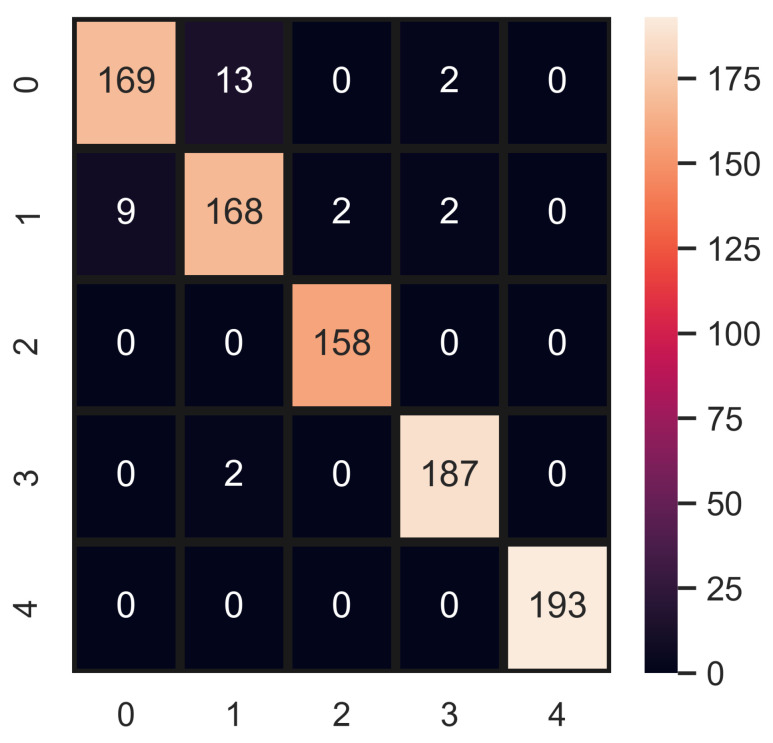
RF confusion matrix using HB features.

A performance comparison of all machine learning models used in this study is illustrated in Figure 6, using different types of features such as original texture and RGB features, augmented texture and RGB features, and hybrid features. It shows that the best performance of the models is obtained when trained on oversampled hybrid features, with RF as the best performer. Figure 7 show the confusion matrix for the best performer RF using machine learning models.

**Figure 8:**
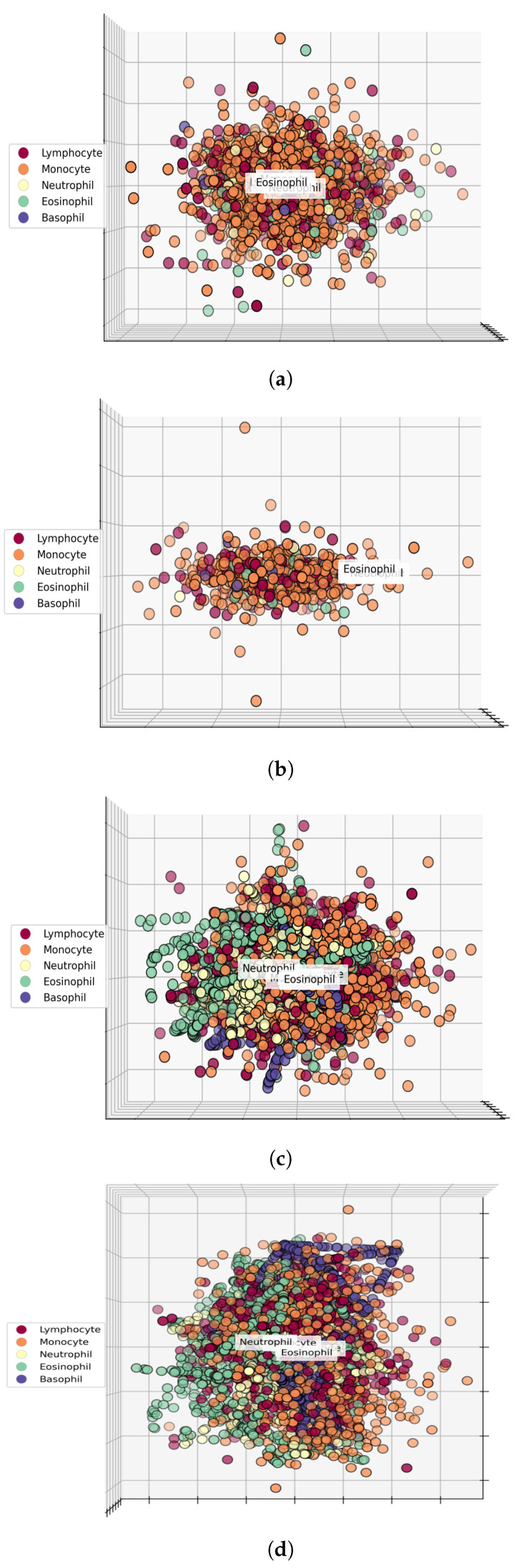
Comparison of original features space and proposed features space. We visualize features in 3D space and show that the features set makes a good correlation with target classes or not. If there will be less overlapping in different target samples, this means that the feature set is good for achieving significant results. We used PCA to convert our features into 3 dimensions, and then illustrated them using a scatter plot. (**a**) Original dataset RGB features, (**b**) Original dataset texture features, (**c**) Hybrid features space at 0°, and (**d**) Hybrid features space at 90°.

Figure 8 shows the comparison of different features, including RGB features, texture features, and hybrid features. It is helpful in understanding the performance of machine learning models when trained on different feature sets. Figure 8a shows the feature space of RGB features, indicating that the distribution of samples of various classes is not well separable, which explains why the performance of machine learning models is not good when using RGB features. The same is true for texture features where samples of different WBC types are mixed, as shown in Figure 8b. However, the feature space of hybrid features on oversampled data, as given in Figure 8c, shows that the sample distribution of WBC types is more separable and distinguishable as compared to both texture and RGB features, which leads to a better performance of the machine learning models.

### 4.4. Performance Analysis of State-of-the-Art Deep Learning Models

**Figure 9:**
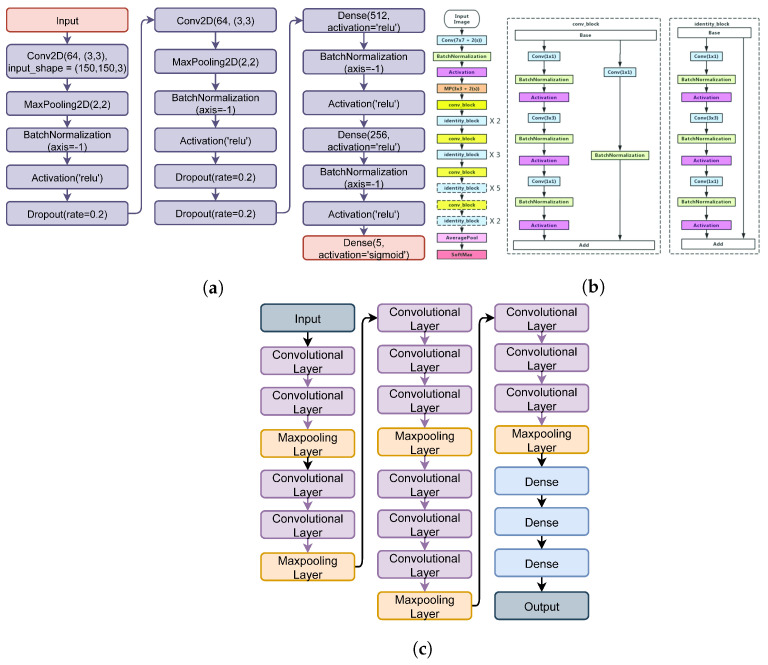
Architecture of the state-of-the-art deep learning models. (**a**) CNN, (**b**) ResNet50 [41], and (**c**) VVG16.

We deployed several deep learning models in comparison with the proposed approach. For this purpose, CNN, VVG16, and ResNet-150 are adopted for WBC-type detection. The architectures of all models are illustrated in Figure 9. Each model consists of a dense layer in the end, with five neurons and a Sigmoid activation function. The models are compiled using the categorical_crossentropy loss function and an Adam optimizer [33]. All models are fitted with 15 epochs, and 15% of the data are used for validation.

**Figure 10:**
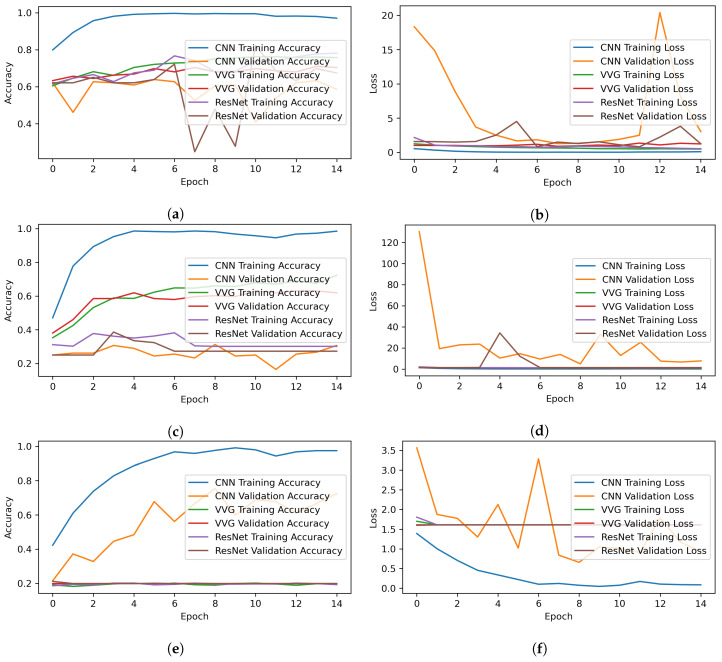
Training and testing per epochs accuracy and loss graphs. (**a**) Accuracy on original dataset, (**b**) Loss on original dataset, (**c**) Accuracy on augmented dataset, (**d**) Loss on augmented dataset, (**e**) Accuracy on over-sampled dataset, and (**f**) Loss on over-sampled dataset.

Figure 10 shows the training and validation loss and accuracy for the CNN and pre-trained models. All models are fitted with 15 epochs, and the results for each epoch are given. The loss and accuracy per epoch are illustrated with respect to the original dataset, augmented dataset, and oversampled dataset. The figure shows that the training and validation accuracy, and loss of CNN are better, as compared to other models.

The performance results of deep learning models are shown in Table 12. The results are presented for three types of datasets, including the original, augmented, and oversampled datasets. However, the handcrafted features are not used for deep learning models such as texture or RGB features, because deep learning models work well with their own extracted features. On the original dataset, pre-trained models are good as compared to CNN, as VVG16 achieves a 0.74 accuracy score with a 0.30 F1 score. The performance of VVG16 is the same in the case of the augmented dataset. In both cases, the original and augmented dataset models are over-fitted on the majority class and show poor performance for the minority class, which results in a poor F1 score. The oversampling technique resolves this issue by providing balanced samples for training, which results in a better performance of CNN, with similar results for the accuracy and F1 score. CNN achieves an accuracy of 0.73 and an F1 score of 0.71 using the oversampled dataset.

### 4.5. Validation of the Proposed Approach

We validate the proposed approach using the k-fold cross-validation technique. From this point of view, a 10-fold cross-validation is applied to all of the machine learning models used in this study with the proposed hybrid features on the oversampled dataset. The results given in Table 13 show that the RF obtains the best performance, with a 0.95 accuracy score and a standard deviation (SD) of ±0.03, followed by the SVM, with a 0.90 accuracy score and ±0.02 SD, which validates the superior performance of RF with the proposed hybrid feature set.

### 4.6. Best Performer Optimization Using Particle Swarm Optimization

We used the swam optimizer for hyperparameter tuning to select the best hyperparameters in comparison with our selected hyperparameters. For this purpose, we used the mealpy 1.0.2 library [42,43]. We imported a particle swarm optimization (PSO) model named BasePSO, and tuned the best performer RF with its two hyperparameters, n_estimators and max_depth. We set upper bound and lower values at 300 and 50, respectively, for both n_estimators and max_depth. We set the epochs value to 2 and the population size to 50 for PSO. The best solution using swam optimizer achieves a 0.94 accuracy as it optimizes the n_estimators value of 273 and max_depth value of 110. Table 14 shows the PSO epoch-wise RF results. It can be observed that RF is good as compared to other models, and the approach is good in terms of accuracy and computational cost.

### 4.7. Performance Comparison with State-of-the-Art Approaches

The performance comparison of the proposed approach is also carried out with existing state-of-the-art approaches. We performed a fair comparison by evaluating all approaches on the dataset that is used. We implemented the models from the selected studies according to their base paper architecture; however, the dataset of the current study is used. For comparison, only those studies are selected which investigate WBC-type classification using medical image datasets. We compared the results of the current study with [15], which proposed a CNN model for WBC classification. Similarly, the study [12] proposed a hybrid model by combining CNN and RNN to perform WBC classification. We selected these studies for comparison because they share the same topic. These studies used an imbalanced medical image dataset. In addition, two additional studies [34,44] that investigate medical image classification are also considered. For a fair comparison, the models presented in these studies are implemented using the dataset used in this study. Performance comparison is carried out in terms of accuracy and computation time. Table 15 shows the performance comparison of selected studies with the current study. It can be observed that the current study outperforms the existing studies both regarding the obtained accuracy and computation time, which shows the supremacy of this approach for WBC-type classification.

### 4.8. Statistical *t*-test

This study also performs a statistical significant *t*-test to show the significance of the proposed approach [45]. We deployed a *t*-test on each approach in comparison with the proposed approach and obtained results in form of acceptance or rejection of the null hypothesis.

- Null Hypothesis ($H_{0}$): if both compared results are statistically equal and there is no significant difference in results, it accepts the null hypothesis.
- Alternative Hypothesis ($H_{a}$): if both compared results are not statistically equal and there is a significant difference in results, it rejects the null hypothesis and accepts the alternative hypothesis.

Table 16 shows the *t*-test results for all cases. The *t*-test gives the *T* score and critical value (CV) score as outputs, and if T≤CV, then it accepts the null hypothesis; else it rejects the null hypothesis. *t*-test rejects the null hypothesis and accepts the alternative hypothesis in all cases when we compared the proposed approach results with other approaches which show that the proposed approach is statistically significant in comparison with other approaches.

## 5. Conclusions

White blood cells are an important part of the immune system, and they protect the body against infections and foreign substances. WBCs consist of different types, and abnormalities in a type of WBC may potentially represent a disease advocating the significance of WBC-type classification. Existing studies are limited by poor accuracy, model over-fitting due to an imbalanced dataset, and classification for a lower number of WBC types. From this perspective, this study presents a hybrid feature set of selective features using Chi2- and SMOTE-based oversampling to alleviate the influence of the above-mentioned problems. In addition, machine learning models are adopted to overcome the limitation of the data-intensive training time of deep learning models and robust results. The experimental results indicate that the feature set of both texture and RGB features from microscopic images selected using Chi2 produces a high accuracy of 0.97 with the RF model. Performance appraisals using k-fold cross-validation, T-statistic test, and comparison with existing state-of-the-art studies show the supremacy of the proposed approach, both in terms of the obtained accuracy and computational complexity. In the future, we intend to perform further experiments using derived features from microscopic data.

## Figures and Tables

**Table 1:** Summary of literature review.

Ref.	Year	Approach	Aim and Results	Dataset
[17]	2018	Pre-trained models ResNet and Inception	WBC detection, Achieved 99.84% and 99.46% accuracy rates with ResNet V1 152 and ResNet 101	WBC microscopic image dataset [19].
[7]	2019	CNN with handcrafted features	WBC detection: CNN achieved 99% accuracy	RBCs, WBCs images dataset [7]. The augmented dataset consists of 2697 cropped images in the `training set’ and 816 cropped images in the `test set’.
[6]	2020	CNN-based models features and ELM model	WBC detection, ELM achieved 96.03% accuracy.	BCCD dataset, Microscopic WBC dataset consists of 12,494 images, including 3120 eosinophils, 3108 lymphocytes, 3095 monocytes, and 3171 neutrophils [20].
[12]	2021	Canonical Correlation Analysis (CCA) applied to (CNN + RNN)	WBC types classification, Model achieved 95.89% accuracy	BCCD dataset [20] and Kaggle dataset
[13]	2021	3D CNN and 3D CNN with attention module	WBC types classification, Model achieved 97.72% accuracy score.	Three independent datasets with 215 patient samples.
[14]	2022	SIFT features with CNN model	WBC detection, Models achieved LISC = 95.84% and WBCis = 97.33% accuracies.	LISC and WBCis datasets [19].
[15]	2022	CNN model	WBC types classification, Model achieved 98.55% accuracy score.	WBC Kaggle dataset [20].
[16]	2022	Multi-level CNN model	WBC types classification, Model achieved 98.4% accuracy score.	Blood Cell Detection (BCD) dataset, Complete Blood Count (CBC) dataset, White Blood Cells (WBC) dataset, Kaggle Blood Cell Images (KBC) dataset, Leukocyte Images for Segmentation and Classification (LISC) dataset
[18]	2022	DenseNet121 model	WBC types classification, Model achieved 98.84% accuracy score.	KBC dataset

**Table 2:** Sample count in the original dataset.

WBC Type	Original	Augmented
Neutrophil	319	194
Lymphocyte	905	-
Monocyte	82	418
Eosinophil	82	405
Basophil	20	447

**Table 3:** Number of features with individual technique.

Feature Type	No. of Features
RGB	67,500
Texture	22,500

**Table 4:** Number of features.

Feature	Total No. of Features	Chi2
RGB	67,500	3000
Texture	22,500	3000
HF	6000	

**Table 5:** Sample count after over-sampling of data.

WBC Type	Original	Augmented	Oversampled
Neutrophil	319	194	905
Lymphocyte	905	-	905
Monocyte	82	418	905
Eosinophil	82	405	905
Basophil	20	447	905

**Table 6:** Target class count in the training and testing sets.

Class	Oversampling Data		
	Training Set	Testing Set	Total
Neutrophil	709	196	905
Lymphocyte	728	177	905
Monocyte	719	186	905
Eosinophil	726	179	905
Basophil	738	167	905
	Original Data		
Neutrophil	249	70	319
Lymphocyte	732	173	905
Monocyte	69	13	82
Eosinophil	59	23	82
Basophil	17	3	20

**Table 7:** Hyperparameters setting for machine learning models.

Model	Hyperparameters	Hyperparameters Tuning
DT	max_depth = 200	max_depth = {2 to 500}
RF	max_depth = 200, n_estimators = 300	max_depth = {2 to 500}, n_estimators = {50 to 500}
KNN	n_neighbors = 5	n_neighbors = {2 to 10}
SVM	Kernel = poly, C = 4.0	Kernel = {poly, linear, sigmoid} C = {1.0 to 5.0}

**Table 8:** Results of machine learning modes on the original dataset.

Feature	Model	Accuracy	Precision	Recall	F1
Texture	DT	0.42	0.19	0.19	0.19
	RF	0.66	0.13	0.20	0.16
	KNN	0.43	0.19	0.23	0.19
	SVM	0.66	0.13	0.20	0.16
RGB	DT	0.49	0.22	0.22	0.22
	RF	0.62	0.36	0.21	0.19
	KNN	0.55	0.23	0.21	0.20
	SVM	0.56	0.25	0.23	0.23

**Table 9:** Results of models on the augmented dataset.

Feature	Model	Accuracy	Precision	Recall	F1
Texture	DT	0.26	0.24	0.24	0.24
	RF	0.44	0.33	0.37	0.35
	KNN	0.25	0.06	0.25	0.10
	SVM	0.33	0.08	0.25	0.12
RGB	DT	0.41	0.39	0.38	0.38
	RF	0.64	0.63	0.57	0.54
	KNN	0.40	0.29	0.34	0.31
	SVM	0.44	0.41	0.41	0.41

**Table 10:** Machine Learning Results on Over-sampled Dataset.

Feature	Model	Accuracy	Precision	Recall	F1
Texture	DT	0.74	0.74	0.74	0.74
	RF	0.92	0.92	0.92	0.92
	KNN	0.77	0.65	0.75	0.68
	SVM	0.77	0.69	0.76	0.70
RGB	DT	0.61	0.61	0.62	0.61
	RF	0.87	0.87	0.87	0.87
	KNN	0.70	0.69	0.70	0.69
	SVM	0.81	0.81	0.81	0.81

**Table 11:** Results of models using the hybrid features from an oversampled dataset.

Feature	Model	Accuracy	Precision	Recall	F1
HF	DT	0.73	0.73	0.73	0.73
	RF	0.97	0.97	0.97	0.97
	KNN	0.78	0.80	0.78	0.72
	SVM	0.89	0.89	0.89	0.89

**Table 12:** Deep learning models performances with each approach.

Sampling	Model	Accuracy	Precision	Recall	F1 Score
Original	CNN	0.58	0.19	0.22	0.20
	VVG16	0.74	0.29	0.31	0.30
	ResNet-50	0.68	0.25	0.29	0.27
Augmention	CNN	0.58	0.19	0.22	0.20
	VVG16	0.74	0.29	0.31	0.30
	ResNet-50	0.68	0.25	0.29	0.27
Oversampling	CNN	0.73	0.74	0.73	0.71
	VVG16	0.20	0.06	0.20	0.07
	ResNet-50	0.20	0.04	0.20	0.06

**Table 13:** Ten-fold cross-validation results of machine learning models using the proposed approach.

Model	Accuracy	SD
DT	0.76	±0.03
KNN	0.79	±0.03
RF	0.95	±0.03
SVM	0.90	±0.02

**Table 14:** RF hyperparameters optimization using PSO.

Epoch	Current Best	Global Best	Runtime	N_estimators	Max_Depth
Epoch 1	0.94	0.94	691	169	253
Epoch 2	0.94	0.94	655	273	110
Best Fitness	0.94				

**Table 15:** Comparison with existing state-of-the-art approaches.

Ref.	Year	Model	Acc.	T (s)
[34]	2021	CNN	46%	1244
[44]	2021	MobileNet+CNN	57%	601
[12]	2021	CNN+RNN	51%	9612
[15]	2022	CNN	72%	1109
This study	2022	RF	97%	34

**Table 16:** Statistically significant *t*-test.

Case	*T*	CV	$H_{0}$
HF + SMOTE V Original	12.125	0.000	Rejected
HF + SMOTE V Augmented	25.698	0.000	Rejected
HF + SMOTE V Over-sampled	16.187	0.000	Rejected

## Data Availability

Not applicable.
